# Priors, population sizes, and power in genome-wide hypothesis tests

**DOI:** 10.1186/s12859-023-05261-9

**Published:** 2023-04-26

**Authors:** Jitong Cai, Jianan Zhan, Dan E. Arking, Joel S. Bader

**Affiliations:** 1 Department of Biomedical Engineering, Johns Hopkins University, Baltimore, MD 21218 USA; 2 Department of Genetic Medicine, Johns Hopkins University, Baltimore, MD 21218 USA

**Keywords:** Genome-wide association studies (GWAS), Genomics, Multiple hypothesis testing, Population genetics, Statistical genetics

## Abstract

**Background:**

Genome-wide tests, including genome-wide association studies (GWAS) of germ-line genetic variants, driver tests of cancer somatic mutations, and transcriptome-wide association tests of RNAseq data, carry a high multiple testing burden. This burden can be overcome by enrolling larger cohorts or alleviated by using prior biological knowledge to favor some hypotheses over others. Here we compare these two methods in terms of their abilities to boost the power of hypothesis testing.

**Results:**

We provide a quantitative estimate for progress in cohort sizes and present a theoretical analysis of the power of oracular hard priors: priors that select a subset of hypotheses for testing, with an oracular guarantee that all true positives are within the tested subset. This theory demonstrates that for GWAS, strong priors that limit testing to 100–1000 genes provide less power than typical annual 20–40% increases in cohort sizes. Furthermore, non-oracular priors that exclude even a small fraction of true positives from the tested set can perform worse than not using a prior at all.

**Conclusion:**

Our results provide a theoretical explanation for the continued dominance of simple, unbiased univariate hypothesis tests for GWAS: if a statistical question can be answered by larger cohort sizes, it should be answered by larger cohort sizes rather than by more complicated biased methods involving priors. We suggest that priors are better suited for non-statistical aspects of biology, such as pathway structure and causality, that are not yet easily captured by standard hypothesis tests.

## Background

Genomics experiments involve testing thousands to millions of hypotheses. In functional genomics and proteomics, each gene or protein usually corresponds to a single test, with 20,000 or more tests required for an RNAseq or proteomics experiment. In human genetics, the number of independent tests accounting for linkage disequilibrium in a single ethnicity is usually assumed to be about 1 million for all but the rarest variants. To maintain a family-wise error rate (FWER) controlled at 0.05, a long-standing approach has been to apply a Bonferroni correction, requiring a single-test* p* value of 0.05 divided by the number of hypotheses tested. This multiple-testing correction from this stringent approach is a burden for identifying genome-wide significant findings.

A robust solution to this problem has been to gather large cohorts of unrelated individuals, particularly for GWAS [1]. While the biological effect of a genetic variant is constant, its corresponding test statistic should be improved with cohort size, yielding greater power to detect. Cohort sizes are limited by the efficiencies of data generation, for example the number of samples that can be genotyped for a typical research budget. Increased DNA sequencing efficiencies permit larger cohorts for RNAseq, and increased DNA synthesis efficiencies reduce the cost of genotyping arrays and permit larger cohorts. Exponential improvements in cost-per-base is a typical model for DNA sequencing and synthesis efficiencies over time. Progress in such exponentially improving fields is often characterized by the doubling time, popularly known as Moore’s law for 1.5–2 year doubling time for the number of transistors on a semiconductor computer chip [2]. Moore’s law analysis applied to the number of DNA bases that can be sequenced or synthesized per dollar has shown a doubling time of approximately 2 years [3]. Of course, sequencing or genotyping costs are only one aspect of a study, and actual cohort sizes may grow at different rates.

Rather than increasing cohort sizes, an alternative approach is to incorporate prior knowledge about functional effects of genes or SNPs. In GWAS, this may increase the power to detect SNPs with true associations or to identify which SNP in a linkage disequilibrium (LD) region is most likely to be the causal variant [4–7]. Other methods incorporate priors based on patterns learned from the data, for example priors for gene-based patterns [8, 9] or phenotype-based patterns [10]. While these methods have value in providing a clearer view of genetic architecture than available through univariate tests, the number of new significant findings has been small [7, 11].

A representative approach incorporated 450 different annotations into GWAS analysis of 18 human traits; the number of loci with high-confidence associations was increased by around 5$\%$ [12]. Despite the intuitive value of incorporating pre-existing biological knowledge, it remains unclear whether this roughly 5% increase in genome-wide significant findings is the best that could be obtained, and additionally whether the increase comes at the cost of false negatives for true positives that lack similar annotations. It is also unclear how this 5% increase compares with the anticipated increase from cohort size alone: given that this more sophisticated analysis itself required 1-2 years of effort, would it have been just as effective to wait a year and then apply simpler methods to a larger cohort?

In this paper, we use theoretical models and derivations to investigate the dependency of power on population size and incorporating priors. We consider an oracular hard prior, which tests a subset of the hypothesis that is guaranteed to include all the true positives. We show analytically that in the limit of small effect sizes and most relevant to genomics studies, population sizes are exponentially more important than priors in determining the power. We then show that given historical trends in cohort sizes, it is nearly impossible for new analytical methods to improve power faster than larger studies that use conventional methods.

## Results

To ground our analysis in improvements in GWAS power that can be anticipated simply due to increases in cohort sizes enabled by increases in DNA analysis efficiencies, we characterized the doubling times for GWAS cohorts and the number of loci detected at genome-wide significance. We then determined analytically the strength of a prior required to give an increase in power equivalent to the power gain from an increased cohort size. We show that an oracular hard prior, which restricts testing to a fraction of tests that still includes every true positive, has difficulty matching the power gain from a typical one-year increase in cohort size. We then show that a non-oracular hard prior, which excludes some true positives from the tested set, often performs worse than tests without a prior at all.

### Doubling times for GWAS cohorts and significant loci

Exponential increases in genotyping and sequencing efficiency have enabled similar increases in GWAS cohort sizes. Larger sample sizes in turn have greater power to detect SNPs with smaller and smaller effects. We quantified increases in cohort sizes and genome-wide significant SNPs through a systematic analysis of GWAS cohorts, traits, and loci as compiled by the GWAS Catalog [13]. Quantification is through an exponential fit, reported as the doubling times of GWAS cohort sizes and detected SNPs in phenotypes with sufficient studies to permit estimation.

The GWAS Catalog contains results for 5123 total studies describing 3034 traits and 126,788 associations that are genome-wide significant, defined as *p* value $5 \times 10^{-8}$ or below, and pruned to report one lead SNP for regions with high linkage disequilibrium. Studies were grouped according to the catalog-assigned disease trait and arranged in chronological order from the oldest to the most recent (see Additional file 1). Cohort sizes were based on the populations reported by the studies. Case-control population sizes were estimated as twice the harmonic mean of the number of cases and controls, a balanced design that should have similar power (see Methods). To avoid analysis of smaller replication studies, studies for a trait were only analyzed if the study identified the largest number of significant loci for that trait as of its publication date. These time-ordered studies for each phenotype were defined as ‘effective studies’. With three effective studies required to permit fitting regression models, we calculated doubling times for cohorts and loci for 49 traits, comprising 282 studies and 23,839 associations (see Additional file 2).

**Fig. 1:**
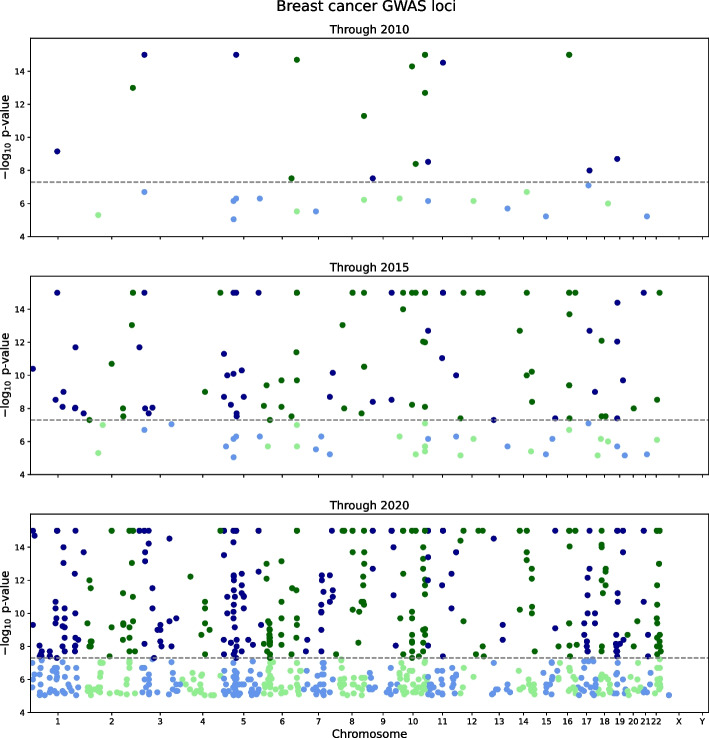
GWAS progress for breast cancer susceptibility cohorts and loci. Manhattan plots depict GWAS findings for breast cancer as of 2010 (top panel), 2015 (middle panel), and 2020 (bottom panel). In each panel, the x-axis represents genomic coordinates to scale, and the y-axis is the $-\log _{10}$ *p* value for a GWAS association with a SNP; a dashed line indicates the genome-wide significance threshold, $p = 5\times 10^{-8}$. The SNP color alternates blue/green by chromosome, with lighter colors for findings below threshold and saturated colors above threshold

Results for a well-studied case-control disease trait, breast cancer susceptibility, demonstrate the progress in identifying genome-significant loci (Fig. 1, data provided in Additional file 3). As of 2010, the largest study had 3659 cases and 4897 controls, an effective cohort size of 8377. Those studies had revealed 19 genome-wide significant loci. As of 2015, the largest effective cohort size was 33,671, and 106 loci had been identified. Studies through 2020 have had an effective cohort size of 138,040 and 411 loci have been identified.

**Fig. 2:**
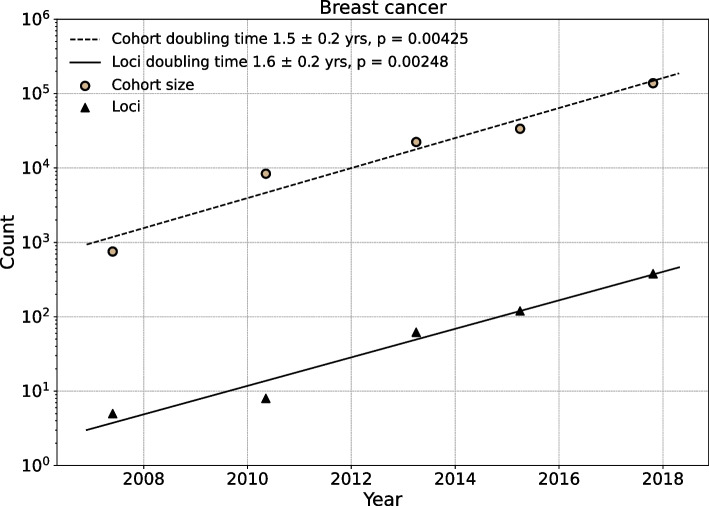
Growth rate of sample size and number of loci for GWAS investigating breast cancer. The scatter plot shows that both the sample size and number of loci increase through time for GWAS investigating breast cancer. The cohort size and numbers of loci increase linearly in log scales during last decade. The x-axis indicates time and the y-axis indicates the counts. Every points is a single study recorded in the GWAS Catalog.

Regression fits for the five breast cancer susceptibility studies estimate the cohort size doubling time to be $\tau _N = 1.5 \pm 0.2$ years, and the genome-wide significant loci doubling time to be $\tau _L = 1.6 \pm 0.2$ years (Fig. 2). This fit, highly significant versus a null hypothesis of no progress, used the log-linear model $\log _2 y = \beta _0 + \beta _1 t$, where *y* is the cohort size or the number of genome-wide significant loci, *t* is the publication date in fractional years, and $\beta _1^{-1}$ is the doubling time. A fit including a quadratic term, $\log _2 y \sim \beta _0 + \beta _1 t + \beta _2 t^2$, did not improve the model for cohort doubling time (*p* = 0.384) or for loci doubling time (*p* = 0.652), justifying the use of an exponential growth model.

**Fig. 3:**
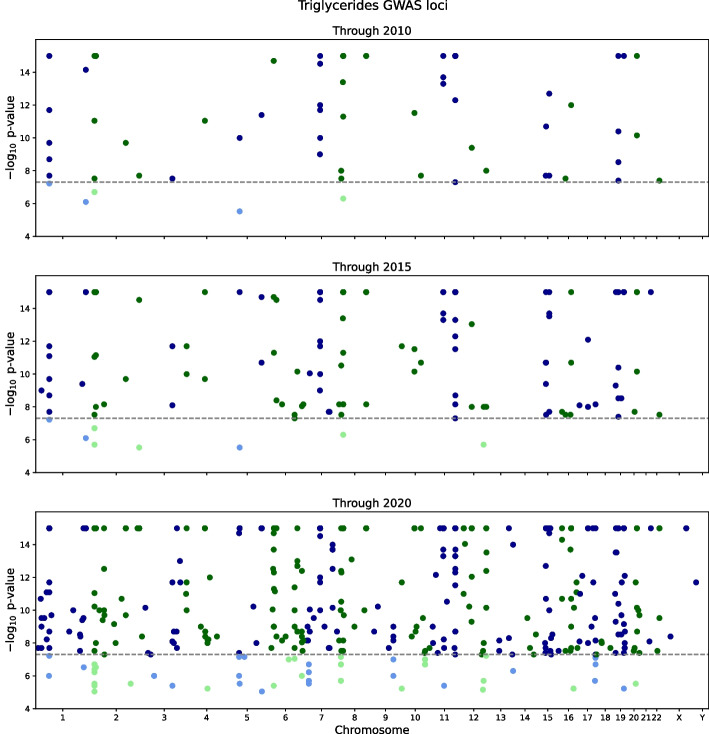
GWAS progress for triglyceride cohorts and loci. Manhattan plots depict GWAS findings for blood triglyceride levels as of 2010 (top panel), 2015 (middle panel), and 2020 (bottom panel). In each panel, the x-axis represents genomic coordinates to scale, and the y-axis is the $-\log _{10}$ *p* value for a GWAS association with a SNP; a dashed line indicates the genome-wide significance threshold, $p = 5\times 10^{-8}$. The SNP color alternates blue/green by chromosome, with lighter colors for findings below threshold and saturated colors above threshold

**Fig. 4:**
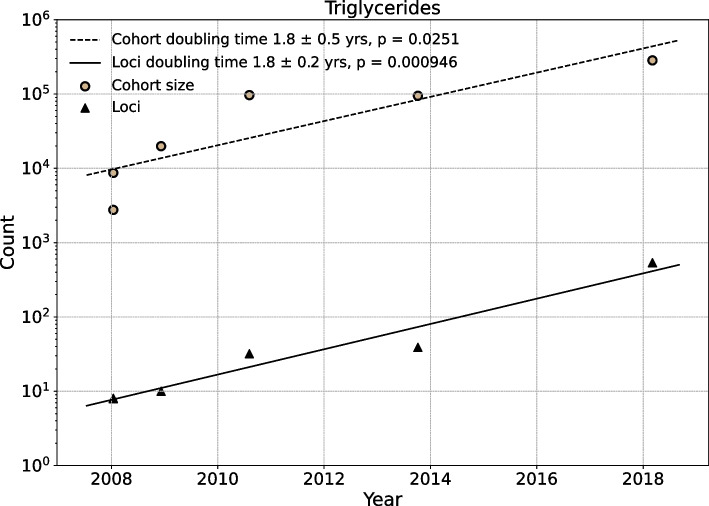
Growth rate of sample size and number of loci for GWAS investigating triglycerides in blood. The scatter plot shows that both the sample size and number of loci increase through time for GWAS investigating triglycerides in blood. The cohort size and numbers of loci increase linearly in log scales during last decade. The x-axis indicates time and the y-axis indicates the counts. Every points is a single study recorded in the GWAS Catalog

Analogous results for a well-studied quantitative trait, blood triglyceride levels, show similar progress from 2010 to 2020 (Fig. 3, data provided in Additional file 4). As of 2010, the largest study had a cohort size of 96,598, and 65 loci had been identified. As of 2020, the largest cohort was 283,251, and 452 loci had been identified. The doubling time for cohorts was estimated at $\tau _N = 1.8 \pm 0.5$ years, and the doubling for loci was estimated at $\tau _L = 1.8 \pm 0.2$ years (Fig. 4).

**Fig. 5:**
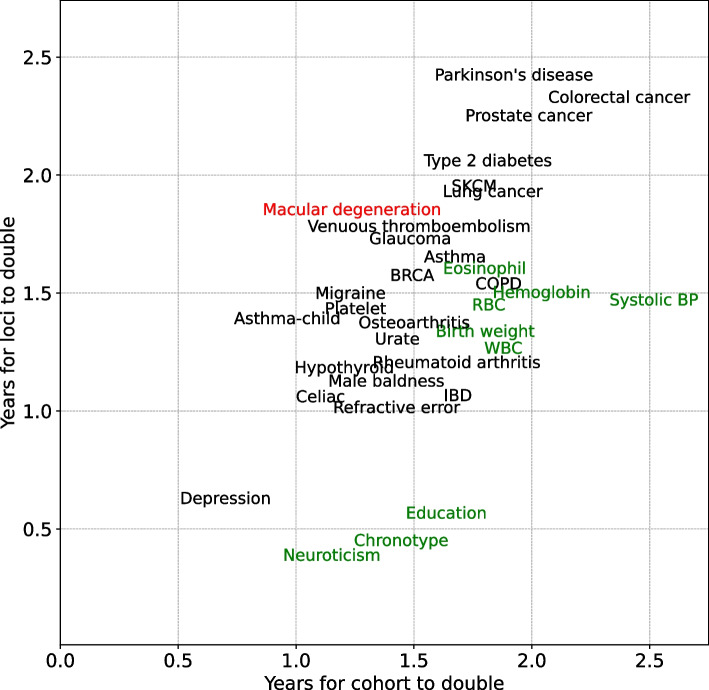
Comparison between years of doubling for loci and cohort in GWAS studies. Each term denotes a trait recorded in the GWAS Catalog. The corresponding x-axis indicates the cohort doubling time for studies investigating that trait, and the y-axis indicates the captured loci doubling time. Terms in black shows that the doubling time for study cohort and loci is approximately the same. Traits that have a significant larger doubling time for cohort than loci are marked in green. Traits with significant larger doubling time for loci are marked in red

We then restricted attention to traits where doubling times could be calculated robustly, with a standard error of 0.5 or smaller, and with at least 10 SNP associations, yielding 33 traits comprising 187 studies and 15,051 associations (see Additional file 2). Results for disease traits where sufficient studies have been reported to estimate doubling times show a general agreement between the doubling time for cohort size and the doubling time for the number of significant loci (Fig. 5). For 23 of the 33 traits analyzed, the null hypothesis of equal doubling times for cohort size and loci cannot be rejected (*p* value = 0.05, two-sided t-test). The only trait for which the number of loci is doubling more slowly than the cohort size is age-related macular degeneration, with a cohort doubling time of 1.2 years and a loci doubling time of 1.8 years. Early studies of this phenotype with small cohorts nevertheless discovered significant loci with large effects. This may explain the subsequent slower rate of discovery.

Traits for which loci are doubling faster than cohort size include neuroticism, chronotype, and education, with cohorts doubling every 1.2 to 1.6 years and loci doubling every 0.4 to 0.6 years. An important aspect noted in a recent educational attainment study is the difficulty in controlling for environmental effects that are correlated with causal variants and overstate the causal effect [14].

Well-defined doubling times imply a power-law for the cumulative distribution $L(R^2)$ of the number of loci with effect size $R^2$ or greater,

$$\begin{aligned} L(R^2) \sim (R^2)^{-\tau _N/\tau _L}. \end{aligned}$$

Equivalence in doubling times leads implies the specific exponent of $-1$,

$$\begin{aligned} L(R^2) \propto 1/R^2. \end{aligned}$$

Algebraic or power-law decay is synonymous with a scale-free distribution and is also known as Zipf’s law. This conjecture is generally consistent with the omnigenic view of complex traits [15].

### Oracular hard priors

The previous results describe why increasing cohort sizes have increasing power to detect additional GWAS loci: the cumulative effect size distribution for many traits has a power-law functional form approximately proportional to $1/R^2$, and a proportional increase in the cohort size *N* can reveal the same proportional increase in genome-wide significant loci.

In this section, we hold the population size fixed and instead present results for oracular hard priors. The term ‘hard’ indicates a frequentist framework in which only hypotheses within a pre-specified subset are tested, and a multiple testing correction is applied according to the number of hypotheses within this subset. The alternative ‘soft’ prior in a Bayesian framework would be a prior probability assigned to each hypothesis, for example arising from predictions of functional impact. The hard prior is valuable in permitting an analytical treatment for general insight into the value of priors.

The ‘oracular’ property indicates that all the hypotheses that in reality fall under the alternative hypothesis are guaranteed to be within the subset selected for testing. This again is a simplification that overestimates any real-world prior, which invariably will create false negatives by not testing some of the hypotheses that really fall under the alternative.

Fewer hypotheses tested corresponds to a reduced multiple-testing burden. This in turn implies a less stringent significance threshold and a greater power to detect positives within the prior region.

**Fig. 6:**
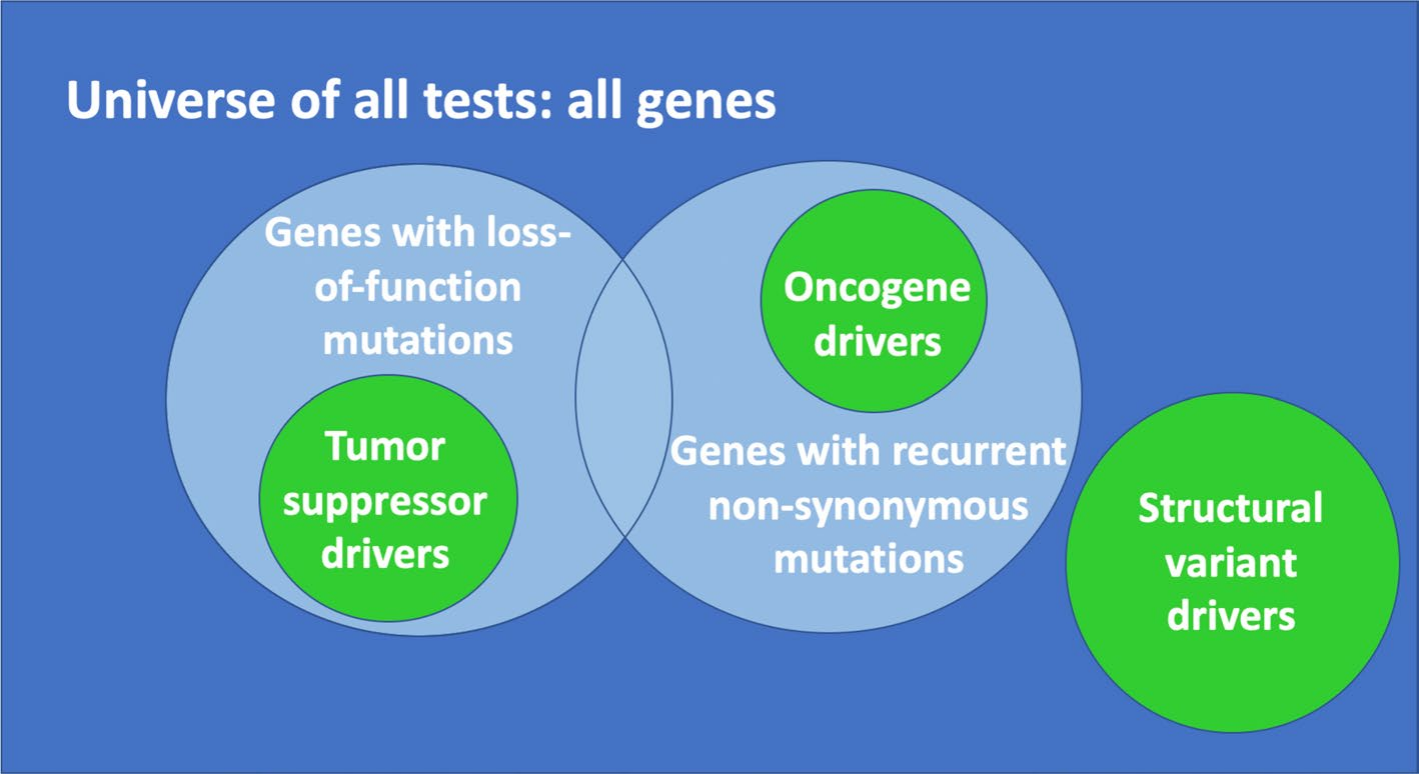
Hard priors in the context of cancer somatic mutations. The universe of all tests (dark blue) includes all identified somatic mutations, regardless of frequency within cancer cells or recurrence across individuals. Hard priors often restrict tests to loss-of-function mutations (light blue) characteristic of tumor suppressors (green) and to recurrent non-synonymous mutations (light blue) characteristic of oncogenes (green). These priors may exclude unanticipated classes of driver mutations, for example structural variants (green) in which copy-number amplifications lead to changes in gene activities that drive cancer. While oracular hard priors have the property that all true findings are within the subset selected for testing, in real-world applications true findings will fall outside the prior and can increase the false-negative rate

Studies of cancer somatic mutations to identify cancer drivers (rather than cancer susceptibility loci) regularly apply hard priors to reduce the testing burden from passenger mutations (Fig. 6). The universe of all possible hypothesis tests includes every somatic mutation identified in a tumor cell versus the germ line, regardless the fraction of tumor cells that carry the mutation or the predicted functional impact of the mutation. Loss-of-function mutations, including deletions, frameshifts, and nonsense mutations, are generally prioritized for testing. Tumor suppressor genes often have these types of mutations. Missense mutations that arise recurrently in independent individuals are also prioritized for testing, with a gain-of-function hypothesis. Oncogenes often have recurrent non-synonymous mutations, or recurrent loss of specific regulatory domains leading to constitutive activity. Mutations that are less likely to have functional consequences, including non-recurrent non-synonymous mutations and mutations in non-protein-coding regions, are often not tested at all. The number of neutral mutations can be orders of magnitude larger than the number of driver mutations [16, 17] Consequently only a small fraction of the observed somatic mutations are tested, considerably reducing the multiple testing burden.

Hard priors for cancer studies are easier to formulate, given the strong functional effects assumed for cancer driver mutations. Similarly, positional cloning studies to identify Mendelian disease loci successfully rely on sequencing to identify germ line mutations with strong functional effects and high penetrance. For GWAS of complex disorders, genetic variants predisposing to disease are less readily identified by strong effects on protein structure or function. Nevertheless, priors that focus attention on genes expressed in a tissue of interest at an appropriate developmental stage, or priors that focus attention on SNPs that are within gene-based boundaries or coincide with regulatory regions, have been discussed and applied.

For a general mathematical framework, we define the strength *S* of a hard prior as the fold-reduction in the number of hypotheses tested: rather than testing all *A* hypotheses, only a subset of size *A*/*S* is tested. The stronger the prior, the larger the value of *S*, and the smaller the multiple testing burden. For a GWAS, a *p* value cutoff of $5\times 10^{-8}$ is usually used for genome-wide tests. A prior with strength $S = 100$, roughly equivalent to testing SNPs in 250 rather 25,000 genes, would change the *p* value cutoff to $5\times 10^{-6}$.

This type of approach can identify some variants that are not identified using a conventional threshold. For example, a breast cancer susceptibility GWAS investigating breast cancer risk identified the SNP rs11571833 in BRCA2 with *p* value of $2\times 10^{-6}$. The BRCA2 gene is a known tumor suppressor contributing to DNA repair. Based on this prior knowledge, the SNP is likely to be a true finding. A strong prior, for example a prior limiting tests to SNPs close to known breast cancer risk factors, would yield a significant finding for rs11571833, which could not be captured as a significant hit with the normal *p*-value cutoff for GWAS studies. This type of prior could suffer from false negatives, however, in excluding most of the genome from testing.

Instead of devising and applying priors, an alternative strategy would be to accumulate larger cohorts for greater power to detect real effects. Larger cohorts and improved priors could of course be pursued simultaneously. Nevertheless, given the 18–24 month doubling time for cohort size and the similar amount of time required to develop and benchmark a new computational method, it is worthwhile to consider the prior strength *S* required to give the same boost in power as simply waiting one to two years for a larger study.

### Power as a function of prior strength and cohort size

Consider two studies with equal power, represented by the normal quantile $z_{I\!I}$ for the false-negative rate for a particular effect size. Typically a power of 80% is desired for a small effect, with $z_{I\!I} = -0.84$. One study has a larger population of size $N_1$ and applies no prior, $S_1 = 1$. This study has a full multiple testing burden, represented by the normal quantile $\zeta$. For GWAS, $\zeta$ corresponds to the quantile for a two-sided *p* value of $5\times 10^{-8}$, or $\zeta = 5.45$. As shown in the methods using a steepest descents approximation for the normal distribution survival function (or the complementary error function),

$$\begin{aligned} \frac{N_1}{N_2} \approx 1 + \frac{2}{\zeta (\zeta - z_{I\!I})} \ln \frac{S_2}{S_1}. \end{aligned}$$

Using the values described above for GWAS, $\zeta (\zeta -z_{I\!I})/2 = 17.2$. The logarithmic term divided by this value is small, permitting the approximation $1 + \epsilon \approx e^{\epsilon }$ with error $\epsilon ^2$. Using this approximation,

$$\begin{aligned} \frac{S_2}{S_1} \approx \left[ \frac{N_1}{N_2} \right] ^{\frac{\zeta (\zeta - z_{I\!I})}{2}} \end{aligned}$$

**Fig. 7:**
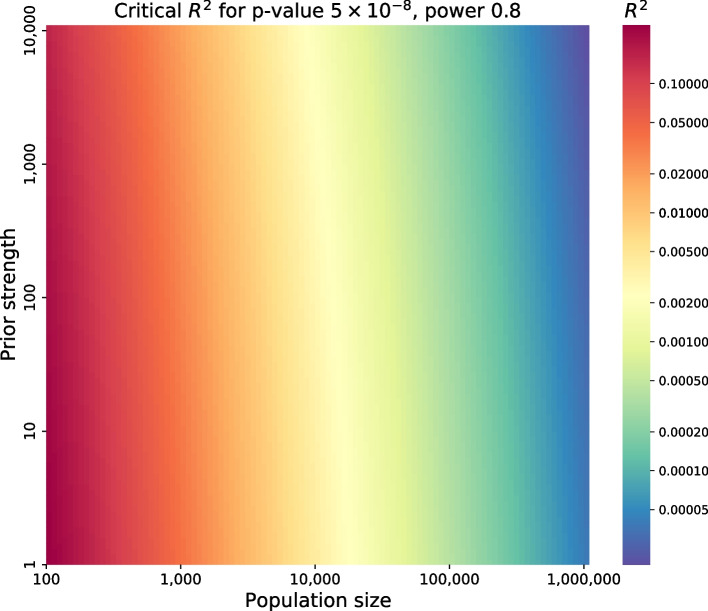
Critical $R^2$ for $p=5\times 10^{-8}$ at power = 0.8 as a function of prior strength and population size. Colors indicate contour lines of equal power to detect an effect in the context of GWAS. Power changes rapidly left-to-right, reflecting the strong dependence of power on population size. Power changes very slowly bottom-to-top, reflecting the weak ability of priors to boost power

The population size is exponentially more important than the prior in determining the critical effect size for a study, defined as the effect size $R^2$ that can be detected with 80% power at significance threshold $\alpha S$, where $\alpha$ is the threshold without a prior and *S* is the prior strength (Fig. 7). The results depicted in Fig. 7 are numerically exact solutions to Eq. 5. The contour lines of equal power as a function of prior strength and population size have a steep slope, slightly steeper in the limit of prior strength 1 (no prior) at the bottom of the figure, and somewhat less steep as the prior strength increases. The numerically exact magnitude of the slope of the contour lines is 17.0 for a prior strength close to 1 and 14.4 for a prior strength of 100, corresponding to 200 genes tested. The analytical approximation yields a slope of $\zeta (\zeta -z_{I\!I})/2 = 17.2$.

**Fig. 8:**
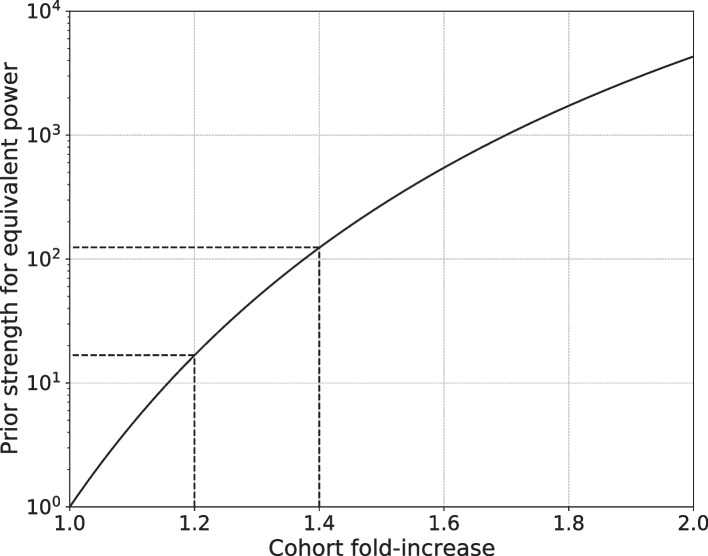
Calibration curve for power and population size. Numerically exact results are shown for the prior strength *S* required to achieve the same power as a simple fold-increase in population size with no prior

The full contour plot of critical $R^2$ is summarized by a numerically exact calibration curve depicting the prior strength required to match the power increase from a larger cohort (Fig. 8). A 1.2-fold larger population could be matched by a prior of strength 16.8, roughly equivalent to restricting tests to genes specific to a tissue of interest. A 1.4-fold larger population could be matched by a prior strength 124.2, roughly equivalent to restricting tests to a pathway of about 100–200 genes. Doubling the cohort size would require a prior strength 4321.5. This would almost certainly violate the oracular property by restricting tests to about 1–10 genes, or to about 500 of the effectively 2 million independent SNPs usually assumed for the GWAS multiple testing burden. This type of prior can be useful for validation studies but would have an unacceptably larger false-negative rate for a discovery study.

### Non-oracular priors

An oracular prior, guaranteed to include every true positive, is an unlikely ideal. More likely is a non-oracular prior with an accuracy *f* defined as the fraction of true positives it includes, with $f = 1$ corresponding to an oracular prior. The power of a test employing a non-oracular prior is the probability of the statistical test itself multiplied by the probability *f* that a particular true positive is actually included in the tested set. Thus, for any specified population size, type I error, and prior strength *S*, a test using a non-oracular prior with $f < 1$ will necessarily have less power than the corresponding oracular power.

For a requested power of $1-\beta$ (with $\beta$ the overall false-negative rate), a statistical test using a non-oracular prior must have power $(1-\beta )/f$, yielding a generalized expression relating the prior strength *S* and prior strength *f* to the cohort size, variance explained, and requested type I and type II error rates (Eq. 11). This relationship can then be used to compare the population size requirement for the non-oracular prior relative to the requirements without a prior (or equivalently, at fixed population size, the effect size that can be discovered at specified family-wise error rate and power). For a given prior strength, consider starting with an oracular prior (accuracy *f* = 1) and decreasing the accuracy. As the accuracy is decreased, the performance of the prior degrades and eventually falls below the performance without a prior. This transition point between using a non-oracular prior and avoiding a prior altogether is readily computed. A second transition occurs when the prior accuracy *f* falls below the requested power $1-\beta$, at which point the test with the non-oracular prior can never yield the requested power regardless of population size.

**Fig. 9:**
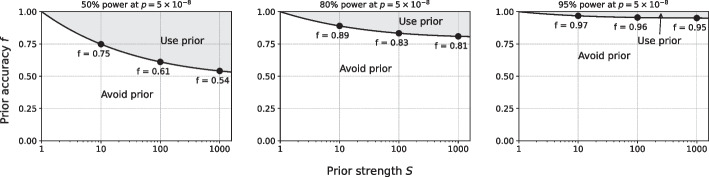
Non-oracular priors for GWAS. Non-oracular priors, with accuracy *f* defined as the fraction of true positives included in the tested set, can perform worse that not using a prior at all. Equal performance (solid line) of a non-oracular prior and of no prior is defined by the $R^2$ that can be detected with 50% power (left), 80% power (middle), or 95% power (right) at $p = 5\times 10^{-8}$ typically used for GWAS. Performance is depicted for non-oracular priors having accuracy *f* from 0% to 100% (equivalent to an oracular prior) and having prior strength *S* from 1 to 1000. Accuracies for equal performance at prior strength 10, 100, and 1000 are shown (labeled points). Above the line, the non-oracular prior performs better; below the line, the non-oracular prior performs worse and should be avoided

Our analyses of non-oracular priors in GWAS are based on a type I error of $5\times 10^{-8}$ and a range of powers of 50% to 95% (Fig. 9). For a typical 80% power requested and a prior strength of 10, corresponding approximately to testing 10% of the genes, an accuracy of 89% is required. If the power strength is 100, corresponding to testing approximately 200 genes, the prior accuracy must be at least 83%.

**Fig. 10:**
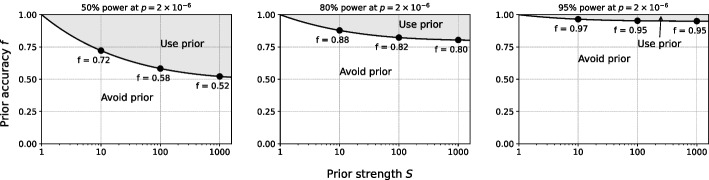
Non-oracular priors for RNAseq. Same as Fig. 9, but for RNAseq, with significance $p = 2\times 10^{-6}$ typically requested. Equal performance (solid line) of a non-oracular prior and of no prior is defined by the $R^2$ that can be detected with 50% power (left), 80% power (middle), or 95% power (right) at $p = 5\times 10^{-8}$. Performance is depicted for non-oracular priors having accuracy *f* from 0% to 100% (equivalent to an oracular prior) and having prior strength *S* from 1 to 1000. Accuracies for equal performance at prior strength 10, 100, and 1000 are shown (labeled points). Above the line, the non-oracular prior performs better; below the line, the non-oracular prior performs worse and should be avoided

While our main focus has been GWAS, similar analyses are readily performed for RNAseq studies. Here, assuming tests of 25,000 genes, the type I error single-test threshold is $2\times 10^{-6}$, and again we consider requested power from 50% to 95% (Fig. 10). Results for RNAseq and GWAS are similar, with a prior accuracy of 88% required for 80% power with 10% of genes tested, and a prior accuracy of 82% for 80% power when only 1% of genes are tested.

For studies with power requested closer to 50%, priors with accuracy of 60% can be effective. Power requests this low are unusual, however, because of potentially large numbers of false negatives. These results suggest that prior accuracies in general must be 90% or higher for non-oracular priors to be considered in practice for genomics studies.

## Discussion

Despite long-standing efforts to exploit prior knowledge to boost the power of GWAS, RNAseq, and other genome-wide tests, including our own efforts to develop and apply gene-based priors for GWAS [8, 9], traditional unbiased univariate tests with a Bonferroni multiple-testing correction or, in RNAseq, a false-discovery rate threshold, are essentially the only methods used in practice. One hypothesis is that genetics researchers performing GWAS do not appreciate the power gains that could be had by using the most recent methods. An alternative hypothesis supported by our findings is that effort is better spent collecting larger cohorts and performing meta-analyses. Supporting the alternative hypothesis are no-free-lunch theorems that prove that priors that improve performance for one class of problems must degrade performance for other classes [18]. Our extension to non-oracular priors highlights the risk a prior with even small inaccuracies can perform worse than an unbiased analysis avoiding priors entirely.

What, then, is the value of priors in GWAS? We are ourselves convinced that developing priors to boost statistical significance for GWAS is not a productive area for future research. We do think, however, that they have an important role in bridging between statistical significance and biological mechanism. While larger cohorts will provide stronger statistical associations, they will not necessarily identify which SNP or SNPs in a linkage disequilibrium block is most likely to be responsible for the observed effect; which gene is affected by the causal SNP; or how many independent effects or different genes are involved. These types of problems are excellent opportunities to develop and use of priors for inference over multiple types of data and existing biomedical knowledge.

## Conclusion

In the context of GWAS, mathematical analyses of standard statistical tests demonstrate that doubling the size of a cohort improves test power more than is possible with any reasonable prior, including oracular priors that are guaranteed to include every true positive in the set of tested hypotheses. In reality, priors are non-oracular and inaccurate, with true positives inevitably left out of the tested set. Whether for GWAS, RNAseq, or other genome-scale hypothesis testing, when even moderate power is a goal, priors can require an unrealistically high accuracy of 90% or more, making it nearly impossible to improve upon performance without a prior.

## Methods

### Empirical data

Datasets were collected from the GWAS Catalog (accessed on June 13, 2020) [13]. An effective sample size was calculated for each study. For studies investigating quantitative traits, the effective sample size was estimated as the number of individuals in the largest cohort described. For case-control studies, the effective sample size was calculated as twice the harmonic mean of the case and control population, estimated to have equal power as follows.

Consider a study with $N_1$ cases and $N_2$ controls. In the context of GWAS, tests for each allele are typically based on $\delta p$, the difference in allele frequency between cases and controls. Denoting these allele frequencies as $p_1$ and $p_2$,

$$\begin{aligned} p_1 = p+\delta p/2, \\ p_2 = p-\delta p/2, \end{aligned}$$

The test statistic is $Q^2$,

$$\begin{aligned} Q^2 = (\delta p)^2 / \textrm{Var}(p_1 - p_2). \end{aligned}$$

Under the null hypothesis, $\delta p = 0$ and $Q^2$ follows a $\chi ^2$ distribution for one degree of freedom.

The estimated allele frequency ${\hat{p}}_1$ is the observed allele count, ${\hat{n}}_1$, divided by the number of chromosomes, $2N_1$. The allele count itself is a binomial random variable with expectation $2N_1p_1$ and variance $2N_1p_1(1-p_1)$. The variance of $p_1$ is therefore

$$\begin{aligned} \textrm{Var}(p_1) = p_1(1-p_1)/2N_1; \end{aligned}$$

similarly,

$$\begin{aligned} \textrm{Var}(p_2) = p_2(1-p_2)/2N_2. \end{aligned}$$

Under the null hypothesis, and for small effect sizes that are typical in GWAS, $\delta p$ is small. Neglecting terms of order $\delta p$,

$$\begin{aligned} \textrm{Var}(p_1-p_2) = \frac{p(1-p)}{2}(\frac{1}{N_1} + \frac{1}{N_2}). \end{aligned}$$

Now suppose that a study involves a population with total size *N*. The variance is minimized with $N_1 = N_2 = N/2$, suggesting our definition of effective population size *N* according to

$$\begin{aligned} \frac{1}{N/2} + \frac{1}{N/2} = \frac{1}{N_1} + \frac{1}{N_2} = \frac{2}{{\bar{N}}}, \end{aligned}$$

where ${\bar{N}}$ is the harmonic mean $2N_1N_2/(N_1 + N_2)$. For the effective population size,

1 $$\begin{aligned} N = 2 {\bar{N}}. \end{aligned}$$

### Traits and effective studies

Each GWAS study may be related to multiple records, each of which demonstrates the significant association between the trait or phenotype investigated by the study and a SNP. These records generally account for linkage disequilibrium by reporting the most significant SNP in a linkage region. The number of associations for each study was counted as the number of associations passing the genome-wide significance threshold of $5\times 10^{-8}$.

The GWAS Catalog assigns a specific trait to each study [13]. We used these trait names defined by the GWAS Catalog to group studies according to trait. For every trait, we then ordered the studies chronologically by publication date. To exclude small replication and validation cohorts, we only retained studies reporting more findings than all previous studies of the same trait. The retained studies were defined as effective studies. Only traits with at least three effective studies (required for log-scale regression fits of an intercept and slope) were kept for further analysis.

Doubling times for cohort sizes and number of associations for each phenotype were estimated as a linear model, $\log _2 {\hat{y}} = \beta _0 + \beta _1 t$, with *y* representing either the cohort size or the number of associations, *t* the publication date with months and days converted to fractional years, and regression coefficients $\beta _0$ and $\beta _1$, corresponding to exponential growth, ${\hat{y}} = 2^{\beta _0} 2^{\beta _1 t}$. The doubling time is $\tau \equiv 1/\beta _1$, and its error is $\sigma _\tau \equiv \sigma _{\beta _1} / \beta _1^2$.

Significance of the exponential fit was assessed by three nested regression models denoted $R_0$, $R_1$, and $R_2$: the null model of no progress, $R_0: \log _2 {\hat{y}} = \beta _0$; the log-linear model of exponential growth, $R_1: \log _2 y = \beta _0 + \beta _1 t$; and a model with more complicated quadratic time dependence, $R_2: \log _2 y = \beta _0 + \beta _1 t + \beta _2 t^2$. Significance of the exponential growth model relative to a null hypothesis of no progress was estimated as the ANOVA *p* value of $R_1$ versus $R_0$. For traits with significant growth, we then assessed the evidence for more complicated time dependence as the ANOVA *p* value of $R_2$ versus $R_1$. Note that for a specific trait, large cohort studies could in principle identify all loci for that trait, yielding a significant model for cohort size growth but no growth in the number of significant loci. The F-statistic of ANOVA test for model sufficiency was calculated as the proportion of extra variation explained by the full model compared to the reduced model. Specifically, to test the time dependence of growth, F-statistic was calculated as the ratio of extra variation explained by $R_1$ compared to $R_0$ and the variation explained by $R_1$ alone; to test whether growth had a more complicated quadratic time dependence, F-statistic was calculated as the ratio of extra variation explained by $R_2$ compared to $R_1$ and the variation explained merely by $R_2$. The corresponding *p* value was then calculated by the F-statistic.

Doubling times for cohort size ($\tau _N$ and error $\sigma _N$ calculated as described above) and number of significant loci ($\tau _L$ and $\sigma _L$ calculated as described above) were compared for a test of the null hypothesis that $\tau _N = \tau _L$. The test statistic $z_\tau$ was defined as $(\tau _N - \tau _L)/\sqrt{ \tau _N^2 + \tau _L^2}$, and *p* values were calculated for a two-sided test of the null hypothesis $z_\tau = 0$. As this analysis was exploratory, we did not correct this test for the number of traits analyzed.

### Relating the significance threshold, power, cohort size, and variance explained for genome-wide tests

We consider tests of association between an observed phenotype or response variable, *y*, and a genotype feature of the data, *x*, testing each of the *A* total genotype features in turn. The *x* and *y* values are assumed to be mean-subtracted scalars for simplicity, and the population size is *N*. Each of the *A* total association tests compares a null model, $M_0$, to an alternative, $M_1$, which for a linear model takes the form

$$\begin{aligned} M_0&:&y \sim \textrm{Norm}(0, \sigma _0^2); \nonumber \\ M_1&:&y \sim \textrm{Norm}(\beta x, \sigma _1^2). \nonumber \end{aligned}$$

One such $M_1$ exists for each feature to be tested. Model parameters are $\Theta _0 = \{ \sigma _0^2\}$ for the null model and $\Theta _1 = \{\beta , \sigma ^2_1\}$ for the alternative model. These models correspond to a null hypothesis $H_0$ and alternative hypothesis $H_1$,

$$\begin{aligned} H_0&:&\beta = 0; \\ H_1&:&\beta \ne 0. \end{aligned}$$

For nested models, the hypothesis test is usually performed by a likelihood ratio test or its equivalent. Assuming independence of the model and data, a test statistic $q^2$ is defined as

2 $$\begin{aligned} q^2 = 2\ln \frac{\textrm{max}_{\Theta _1}\textrm{L}(\Theta _1 \mid y)}{\textrm{max}_{\Theta _0}\textrm{L}(\Theta _0 \mid y)}. \end{aligned}$$

As usual, we define ${\hat{\Theta }} \equiv {\arg \max }_\Theta \textrm{L}(\Theta \mid y)$. For normally distributed *y*, with $\beta \equiv 0$ for $M_0$ and possibly non-zero for $M_1$, the log-likelihood is

$$\begin{aligned} \ln \textrm{L}(\Theta \mid y) = -\frac{N}{2} \ln (2 \pi \sigma ^2) - \frac{1}{2 \sigma ^2} \sum _{i=1}^{N}(y_i-\beta x_i)^2. \end{aligned}$$

For the maximum likelihood parameters, $\ln {\text{L}}(\hat \Theta \mid y) = - (N/2)\ln (2\pi {\hat \sigma ^2}) - (1/2)$. Standard results are

$$\begin{aligned} {\hat{\sigma }}_0^2= & {} \frac{1}{N} \sum _{i=1}^N y_i^2 \\ {\hat{\beta }}= & {} \frac{\sum _{i=1}^N x_i y_i}{ \sum _{i=1}^N x_i^2 }\\ {\hat{\sigma }}_1^2= & {} \frac{1}{N} \sum _{i=1}^N (y_i - \beta x_i)^2 \\ q^2= & {} N \ln \frac{ {\hat{\sigma }}_0^2 }{ {\hat{\sigma }}_1^2 }. \end{aligned}$$

Again following standard definitions, the residual sums of squares for $M_0$ is $\textrm{RSS}_0 =$ $N {\hat{\sigma }}_0^2$ and for $M_1$ is $\textrm{RSS}_1 =$ $N {\hat{\sigma }}_1^2$. The fraction of variance explained by $M_1$ is $(\textrm{RSS}_0-\textrm{RSS}_1)/\textrm{RSS}_0$, defined as $R^2$. The test statistic $q^2$ can be expressed in terms of $R^2$ as

3 $$\begin{aligned} q^2 = N \ln \frac{ \textrm{RSS}_0}{\textrm{RSS}_1} = N \ln \left( 1+ \frac{R^2}{1-R^2} \right) . \end{aligned}$$

According to Wilks’s Theorem, when sample size $N \rightarrow \infty$ under the null hypothesis, $q^2$ is a random variable distributed as $\chi ^2_1$, or more generally as a $\chi ^2_d$ random variable where the null model is nested inside an alternative model with *d* additional parameters [19]. Under the alternative hypothesis, for small $R^2$ typical of GWAS, $q^2$ is distributed as a non-central $\chi ^2$ with non-centrality parameter $q_1^2$,

4 $$\begin{aligned} q_1^2 = N \frac{R^2}{1-R^2}. \end{aligned}$$

To control the type I error (false-positive rate) at family-wise error rate (FWER) $\alpha$, the Bonferroni method requires a single-test *p* value of $\alpha /A$ for *A* total tests. Let the quantile of the uniform normal distribution corresponding to a two-tailed test at this stringency be $z_I$. More formally, if $\Phi (z)$ is the cumulative lower-tail probability distribution for standard normal random variable *z*, then $\Phi (-z_I) = \alpha /2A$. With true effect $q_1$ defined as the positive value $\sqrt{q_1^2}$, the power is $\Phi ( q_1 - z_I)$. Suppose the type II error rate is $\beta$, and $z_{I\!I}$ is the corresponding quantile $\Phi (z_{I\!I})=\beta$. From the definition of power, $\Phi (q_1 - z_I) = 1 - \beta$, implying $q_1 - z_1 = -z_{I\!I}$, or $z_I - z_{I\!I} = q_1$. Therefore,

5 $$\begin{aligned} (z_I - z_{I\!I})^2 = N \frac{R^2}{1-R^2} . \end{aligned}$$

This key expression relates the type I error (false-positive rate), the type II error (false-negative rate or complement of power), the population size *N*, and the effect size $R^2$.

### Effect size distribution

In the limit of small effect size, $R^2 \ll 1$, and fixed type I and type II error, the effect size and population size are inversely related,

$$\begin{aligned} (z_I - z_{I\!I})^2 = NR^2. \end{aligned}$$

This relationship, together with doubling times, implies a functional form for the number of loci with effect size $R^2$ or larger, defined as $L(R^2)$. As before, define $\tau _N$ as the doubling time for cohorts and $\tau _L$ as the doubling time for loci. The effect size that can be discovered at a specified type I error is approximately equal to the effect size at 50% power,

$$\begin{aligned} R^2_N \approx z_I^2/N. \end{aligned}$$

After *t* years, cohort size increases from an initial value $N_0$ to a final value $N_t = N_0 2^{t/\tau _N}$. Similarly, the number of loci discovered increases from $L_0$ to $L_0 2^{t/\tau _L}$. The number of loci at the end is also equal to the the number of loci with effect size greater than or equal to $z_I^2/N(t)$,

$$\begin{aligned} L_0 2^{t/\tau _L} = L(z_I^2 N_0^{-1} 2^{-t/\tau _N}). \end{aligned}$$

This relationship is satisfied in turn by a power-law dependence of $L(R^2)$ on its argument,

$$\begin{aligned} L(R^2) \sim (R^2)^{-\tau _N/\tau _L}. \end{aligned}$$

The probability density has the form of the derivative of the cumulative probability, and thus well-defined doubling times imply an effect-size probability distribution $\rho (R^2)$ with functional form

$$\begin{aligned} \rho (R^2) \sim (R^2)^{-(\tau _L + \tau _N)/\tau _L}. \end{aligned}$$

### Oracular hard priors

We consider an idealized prior in which only hypotheses corresponding to a faction 1/*S* of the total are tested, with an oracular property that all known positives lie within the selected subset. Larger *S* corresponds to a stronger prior. For 20,000 gene-based tests, testing 10% of the total corresponds to $S = 10$, and testing 20 genes corresponds to $S = 1000$. Realistically, priors stronger than $S = 100$, corresponding to 200 genes tested, are unlikely.

The effect of a hard prior is to reduce the multiple-testing burden. To maintain FWER $\alpha$, each two-tailed test is performed at stringency $S\alpha /2A$ rather than $\alpha /2A$. This reduces the quantile $z_I$ required for significance and increases the power to detect an association with a smaller effect $R^2$. Equivalently, Eq. 5 can be solved for $R^2$ to calculate the critical effect size to achieve desired power at stated type I error,

6 $$\begin{aligned} R^2 = \frac{(z_I - z_{I\!I})^2}{N + (z_I - z_{I\!I})^2}. \end{aligned}$$

In other words, given a fixed single test *p* value $\alpha$, *A* total tests and a prior strength of *S*, Eq. 6 can be used to calculated the increases of detectable critical effect size brought by the prior. Results were generated as numerically exact solutions to these equations.

As shown below (see Results), priors were much less effective than increased population sizes in improving power. We developed analytical approximations that helped us understand the reasons for our results. A steepest descents approximation relates the quantile $z > 0$ to its upper-tail area $\epsilon$,

$$\begin{aligned} \epsilon&= (2\pi )^{-1/2} \int _z^\infty du\, e^{-u^2/2} \nonumber \\&= (2\pi )^{-1/2} e^{-z^2/2} \int _z^\infty du\, e^{-(u+z)(u-z)/2} \nonumber \\& \approx (2\pi )^{-1/2} e^{-z^2/2} \int _z^\infty du\, e^{-2z(u-z)/2} \nonumber \\&= (2\pi )^{-1/2} e^{-z^2/2} \int _z^\infty du\, e^{-z(u-z)} \nonumber \\&= \frac{1}{\sqrt{2\pi }z}e^{-z^2/2}. \end{aligned}$$

Equivalently,

$$\begin{aligned} z^2 \approx - 2 \ln [ \sqrt{2 \pi } z \epsilon ]. \end{aligned}$$

In terms of the quantile $z_I$ for prior strength *S* and a two-tailed test, we have approximately

7 $$\begin{aligned} z_I^2 \approx - 2 \ln [ \sqrt{2\pi } z_I S \alpha / A]. \end{aligned}$$

Define $\zeta$ as the value of $z_I$ for no prior, $S = 1$, with $\Phi (-\zeta ) = \alpha /2A$ and

8 $$\begin{aligned} \zeta ^2 \approx -2\ln (\sqrt{2\pi } \zeta \alpha /A). \end{aligned}$$

For GWAS with a *p* value threshold of $5\times 10^{-8}$, $\zeta = 5.45$ and $\zeta ^2 = 29.7$. Because the dependence of Eq. 7 on $\ln z$ is weak, we replace $\ln z$ with $\ln \zeta$,

$$\begin{aligned} z_I^2 \approx - 2 \ln [ \sqrt{2\pi } \zeta S \alpha / A] \approx \zeta ^2 - 2\ln S = \zeta ^2(1-2 \zeta ^{-2} \ln S). \end{aligned}$$

Keeping terms of order $1/\zeta$,

$$\begin{aligned} z_I\approx & {} \zeta (1 - \zeta ^{-2} \ln S) \nonumber \\ z_I - z_{I\!I}\approx & {} \zeta - z_{I\!I} - \zeta ^{-1} \ln S \nonumber \\ (z_I - z_{I\!I})^2\approx & {} (\zeta - z_{I\!I})^2 - \frac{2(\zeta - z_{I\!I})}{\zeta } \ln S \nonumber \\= & {} (\zeta - z_{I\!I})^2\left[ 1 - \frac{2}{\zeta (\zeta - z_{I\!I})}\ln S\right] . \end{aligned}$$

According to Eq. 5, the critical effect size depends only on the ratio $(z_I - z_{I\!I})^2/N$. Consider two scenarios with equal critical effect size, one with population size $N_1$ and prior strength $S_1$, and the second with population size $N_2$ and prior strength $S_2$. For these to have equal critical effect size,

$$\begin{aligned} (\zeta - z_{I\!I})^2\left[ 1 - \frac{2}{\zeta (\zeta - z_{I\!I})}\ln S_1 \right] /N_1 \approx (\zeta - z_{I\!I})^2\left[ 1 - \frac{2}{\zeta (\zeta - z_{I\!I})}\ln S_2 \right] /N_2. \end{aligned}$$

Cancelling constant terms $\zeta - z_{I\!I}$ and noting that $2 \zeta ^{-1} (\zeta - z_{I\!I}) \ln S$ is small,

$$\begin{aligned} \frac{N_1}{N_2}\approx & {} \left[ 1 - \frac{2}{\zeta (\zeta - z_{I\!I})}\ln S_1 \right] / \left[ 1 - \frac{2}{\zeta (\zeta - z_{I\!I})}\ln S_2 \right] \nonumber \\\approx & {} \left[ 1 - \frac{2}{\zeta (\zeta - z_{I\!I})}\ln S_1 \right] \times \left[ 1 + \frac{2}{\zeta (\zeta - z_{I\!I})}\ln S_2 \right] \nonumber \\\approx & {} 1 + \frac{2}{\zeta (\zeta - z_{I\!I})} \ln \frac{S_2}{S_1}. \end{aligned}$$

The dependence on population size is linear, whereas the dependence on prior strength is logarithmic. Equivalently, population size is exponentially more important that prior strength. Again for GWAS with $z_{I\!I}$ selected for 80% power, $\zeta (\zeta -z_{I\!I})/2 = 17.15$, and only a small fractional population increase is required to obtain the equivalent power increase for a strong prior. In the equation above, the logarithmic term divided by $\zeta (\zeta -z_{I\!I})/2$ is small, permitting the approximation $1 + \epsilon \approx e^{\epsilon }$ with error $\epsilon ^2$. Using this approximation,

$$\begin{aligned} \frac{N_1}{N_2} \approx \exp \left[ \frac{2}{\zeta (\zeta - z_{I\!I})} \ln \frac{S_2}{S_1} \right] \approx \left[ \frac{S_2}{S_1} \right] ^{\frac{2}{\zeta (\zeta - z_{I\!I})}} \end{aligned}$$

An extremely strong prior with $S_2 = 1000$, with effectively only 20 genes selected for testing, can be matched by a population increase of about 40%.

Contours of *N* and *S* with equal critical effect size can be estimated by returning to the approximate result

$$\begin{aligned} N R^2/(1-R^2) \approx (\zeta - z_{I\!I})^2[1 - \frac{2}{\zeta (\zeta - z_{I\!I})}\ln S]. \end{aligned}$$

Noting that for small $\epsilon$, $1 + \epsilon \ln S \approx S^{\epsilon }$, contours are given by

9 $$\begin{aligned} N \approx S^{-2 / \zeta (\zeta - z_{I\!I})} (\zeta - z_{I\!I})^2 (1-R^2) / R^2. \end{aligned}$$

On a log-log plot of $\log S$ versus $\log N$, these contours would have steep negative slope equal to $-\zeta (\zeta - z_{I\!I})/2$.

### Non-oracular priors

A non-oracular prior has an accuracy *f* defined as the fraction of true positives contained within the tested set. In the limit $f \rightarrow 1$, the tested set contains all the true positives and the prior is oracular. The prior strength *S* retains its definition as the ratio of the total number of possible tests, *A*, to the tested set with size *A*/*S*. We again define $\Phi (z)$ as the lower-tail area for standard normal quantile *z* and $\Phi ^{-1}$ as its inverse, with $z_\epsilon \equiv \Phi ^{-1}(1-\epsilon )$ for $0< \epsilon < 1$. Without a prior, $z_I =$ $z_{\alpha /2A}$, and with the prior, $z_I =$ $z_{\alpha S/2A}$ as before.

With overall false-negative rate $\beta$, power $1-\beta$, and no prior, $z_{I\!I} =$ $z_{1-\beta }$. With a prior, however, the final power is the product of the power of the statistical test, $(1-\beta ')$ and the prior accuracy *f*. Therefore, the statistical test must have power $(1-\beta )/f$ for the overall power to be $1-\beta$. The value of $z_{I\!I}$ for a non-oracular test is $z_{(1-\beta )/f}$. Note, however, that there are no solutions if the requested power $1-\beta$ is greater than the prior accuracy *f* (which would correspond to a lower-tail area $\ge 1$). These results yield a generalized version of Eq. 5,

10 $$\begin{aligned} (z_{\alpha S / 2A} - z_{(1-\beta )/f})^2 = N \frac{R^2}{1-R^2}, \end{aligned}$$

with $S = f = 1$ for no prior, $S > 1$ with $f=1$ for an oracular power, and $S> 1$ and $f<1$ for a non-oracular prior. For a given family-wise error rate $\alpha$ and population size *N*, the effect size $R^2$ that can be discovered with requested power $1-\beta$ depends on $(z_I - z_{I\!I})^2$. Using non-oracular prior has equivalent performance to using no prior when

11 $$\begin{aligned} (z_{\alpha S / 2A} - z_{(1-\beta )/f})^2 = (z_{\alpha / 2A} - z_{1-\beta })^2. \end{aligned}$$

When the left-hand side of Eq. 11 is smaller (corresponding to a smaller population size required for given effect size $R^2$), the non-oracular prior is preferred. When the right-hand side is smaller, an unbiased test without a prior is preferred.

## Supplementary information

Additional file 1. GWAS Catalog summary: A summary of the traits and studies from the GWAS Catalog that were analyzed.

Additional file 2. GWAS trait summary: A summary of the 49 traitsin the GWAS cCatalog with at least 3 effective studies, permittingestimation of doubling times for cohorts and loci.

Additional file 3. Breast cancer GWAS significantassociations: Data table used to generate Fig. 1.

Additional file 4. Triglycerides GWAS significantassociations: Data table used to generate Fig. 3.

## Data Availability

The datasets generated and/or analysed during the current study are available from a GitHub repository, https://github.com/joelbaderlab/hardprior_2023. This repository also includes all software under a permissive open source license.

## Abbreviations

GWAS: Genome-wide association study (or studies)
SNP: Single-nucleotide polymorphism
